# Vocal fold restoration after scarring: biocompatibility and efficacy of an MSC-based bioequivalent

**DOI:** 10.1186/s13287-023-03534-x

**Published:** 2023-10-21

**Authors:** Mikhail Svistushkin, Anastasia Shpichka, Polina Bikmulina, Alexey Fayzullin, Anna Zolotova, Nastasia Kosheleva, Liliya Selezneva, Boris Shavkuta, Viktoria Lobacheva, Anna Nikiforova, Peter Kochetkov, Svetlana Kotova, Svetlana Starostina, Anatoly Shekhter, Andrey Svistunov, Valeriy Svistushkin, Peter Timashev

**Affiliations:** 1 Department for ENT Diseases, Sechenov University, Moscow, Russia; 2 Institute for Regenerative Medicine, Sechenov University, Moscow, Russia; 3 Chemistry Department, Lomonosov Moscow State University, Moscow, Russia; 4 FSBSI Institute of General Pathology and Pathophysiology, Moscow, Russia; 5 Department of Polymers and Composites, N.N. Semenov Federal Research Center for Chemical Physics, Russian Academy of Sciences, Moscow, Russia; 6 Sechenov University, Moscow, Russia; 7 World-Class Research Center “Digital Biodesign and Personalized Healthcare”, Sechenov University, Moscow, Russia

**Keywords:** Vocal folds, Mesenchymal stromal cells, Fibrin, Tissue engineering, Regenerative medicine, Scarring, PEG-fibrin, Bioequivalent, In vivo

## Abstract

**Background:**

There is growing interest to application of regenerative medicine approaches in otorhinolaryngological practice, especially in the framework of the therapy of vocal fold (VF) scar lesions. The used conservative and surgical methods, despite the achieved positive outcomes, are frequently unpredictable and do not result in the restoration of the VF’s lamina propria’s structure, which provides the mechanical properties necessary for vibration. In this connection, the aim of this study was to ascertain the safety and efficacy of a bioequivalent in the treatment of VF scars using a rabbit model of chronic damage.

**Methods:**

The bioequivalent consisted of a hydrogel system based on a PEG-fibrin conjugate and human bone marrow-derived MSC. It was characterized and implanted heterotopically into rats and orthotopically into rabbits after VF scar excision.

**Results:**

We showed that the fabricated bioequivalent consisted of viable cells retaining their metabolic and proliferative activity. While being implanted heterotopically, it had induced the low inflammatory reaction in 7 days and was well tolerated. The orthotopic implantation showed that the gel application was characterized by a lower hemorrhage intensity (*p* = 0.03945). The intensity of stridor and respiratory rate between the groups in total and between separate groups had no statistically significant difference (*p* = 0.96 and *p* = 1; *p* = 0.9593 and *p* = 0.97…1, respectively). In 3 days post-implantation, MSC were detected only in the tissues closely surrounding the VF defect. The bioequivalent injection caused that the scar collagen fibers were packed looser and more frequently mutually parallel that is inherent in the native tissue (*p* = 0.018). In all experimental groups, the fibrous tissue’s ingrowth in the adjacent exterior muscle tissue was observed; however, in Group 4 (PEG-Fibrin + MSC), it was much less pronounced than it was in Group 1 (normal saline) (*p* = 0.008). The difference between the thicknesses of the lamina propria in the control group and in Group 4 was not revealed to be statistically significant (*p* = 0.995). The Young’s modulus of the VF after the bioequivalent implantation (1.15 ± 0.25 kPa) did not statistically significantly differ from the intact VF modulus (1.17 ± 0.45 kPa); therefore, the tissue properties in this group more closely resembled the intact VF.

**Conclusions:**

The developed bioequivalent showed to be biocompatible and highly efficient in the restoration of VF’s tissue.

**Supplementary Information:**

The online version contains supplementary material available at 10.1186/s13287-023-03534-x.

## Introduction

Scar lesions of the vocal folds (VF) are an important and complicated problem in otorhinolaryngology, which results in a persistent impairment of the vocal function. The current treatment tactics for VF scars includes both conservative and surgical approaches. The conservative treatment is a first line of choice, as a rule; speech therapy shows good results for small scars and may be utilized both alone and as a part of a complex treatment [1, 2]. The surgical approach is recommended no earlier than 6 months after the scar formation [1], and a large number of techniques are used, including endolaryngeal injection and implantation medializations, medialization thyroplasty, epithelium separation from the scar, intramucosal implantations of autologous tissues and synthetic preparations and their different combinations [1–13]. The use of surgical techniques allows reducing the air loss and fatigue during phonation, and, to a certain extent, improving the VF’s viscoelastic characteristics. However, in spite of the diversity of these methods, their result is frequently unpredictable and essentially limited, since there exists no method capable of repairing the structure of the VF’s lamina propria which provides the mechanical properties necessary for vibration [3, 14, 15].

Therefore, great interest arose to the techniques and approaches of regenerative medicine for the development of an efficient way of treatment for VF scar lesions [15–19]. In experimental studies, positive effects of cells from various sources have been demonstrated, such as the reduction in general fibrosis signs, level of inflammation, thickness of the scar tissue, restoration of the extracellular matrix architecture and the tissues’ mechanical parameters [20–24]. To date, the first clinical trial with the use of bone marrow-derived and adipose tissue-derived MSC has been performed [25, 26].

Nevertheless, the question of carriers for cells in an VF implant remains open. Their application was shown to enhance the final result of the VF restoration due to the decrease in the number of lost cells and increase in their viability upon injection, etc.[27]. Since, in most cases, the main mechanism of the administered cells’ action is based on paracrine effects, their “preservation” in the early times after the surgery may be crucial [28–30]. In the majority of studies, a successful application of hyaluronic acid and collagen as carriers has been shown [31–34]. However, considerable interest is also aroused in fibrin, which is used as a hemostatic aid and in the reconstructive surgery of airway stenoses, and represents an alternative to the suture attachment of microflaps of the VF mucosa [1, 35]. Park et al. found that fibrin application for encapsulation of adipose tissue-derived MSC provided their sustained high proliferative activity and more intense expression of elastin as compared to gels based on collagen and hyaluronic acid [36]. Besides, Long et al. showed that the use of bioequivalents based on fibrin and adipose tissue-derived MSC in the damaged VF allowed achieving the vibrational and viscoelastic properties close to those of the native tissues [37].

One of the important advantages of fibrin is a possibility of its targeted modification that allows one to vary the material’s rate of degradation and to form an optimal microenvironment for cells. Indeed, we have earlier shown that such hydrogel systems, in particular, those based on fibrin conjugates with polyethylene glycol (PEG), promoted cells’ high proliferative and migration activity, as well as tubulo- and lumenogenesis, due to the adapted structure [38–40]. Thus, the aim of this study was to ascertain the safety and efficacy of a bioequivalent consisting of a hydrogel system based on a PEG-fibrin conjugate and human bone marrow-derived MSC, in the treatment of VF scars, using a rabbit model of chronic damage.

## Materials and methods

### Cell culture

The primary culture of human bone marrow-derived MSC was isolated from a biopsy material harvested from healthy donors after signing an informed consent, provided by the Sechenov University Biobank. The separation of cells was performed according to the earlier described protocol [41]. Cells were cultured in the standard conditions at the temperature of 37 °C and 5% CO_2_ in a complete growth medium of the following composition: DMEM/F12 (1:1) with the addition of glutamine (2 mM L, Biolot, Russia), gentamycin (50 µg/mL, PanEco, Russia), insulin-transferrin-selenium (1:100) (Biolot, Russia), bFGF (20 ng/mL, Prospec, Israel), and FBS (10%, HyClone, USA). The medium was replaced every 2–3 days. No older than the 4th passage cells were used.

To confirm that the isolated cells belonged to the MSC population, the surface markers were characterized by immunofluorescence flow cytometry, and the ability to adipo-, osteo- and chondrogenic differentiation was assessed. Immunophenotyping was performed in accordance with the standard panel of surface markers (CD11b, CD44, CD19, CD29, CD34, CD45, CD73, CD90, CD105) using a SH800S microfluidic sorter (Sony Biotechnology, USA). For the analysis, cells were detached from the plastic surface and stained with the mixture of antibodies for 15 min in the dark. Then, the suspensions were washed in a phosphate buffer, and the population analysis was performed (stopped upon reaching 20,000 events for each sample). As a control, unstained cell suspensions were used, and the isotypic control was also present. To test the ability of differentiation into the three lineages, cells were seeded on the plastic surface and cultured up to reaching a monolayer. Then, the standard culture media were replaced with induction media: osteogenic (StemPro™ Osteogenesis Differentiation Kit, ThermoFisher Scientific, USA), chondrogenic (StemPro™ Chondrogenesis Differentiation Kit, ThermoFisher Scientific, USA), and adipogenic (StemPro™ Adipogenesis Differentiation Kit, ThermoFisher Scientific, USA). The medium replacement was then performed regularly (1 replacement every three days) for 21 days of cell differentiation. As a negative control, cells cultured in the complete growth medium were used. Then, the samples were stained to confirm the differentiation: with Oil Red O for adipogenic differentiation (Sigma-Aldrich, Germany), with Alizarin Red S for osteogenic differentiation (Sigma-Aldrich, Germany), and with Alcian Blue for chondrogenic differentiation (Sigma-Aldrich, Germany). Before staining, all the samples were preliminarily washed in the phosphate-saline buffer from the residual culture medium and fixed in PFA (4%, pH 6.9, Sigma-Aldrich, Germany) for 20 min at 4 °C. To analyze the adipogenic differentiation, the samples were washed with 60% isopropanol and dried on air for 5 min. Then, 0.2% Oil Red O prepared with 60% isopropanol was added, and the samples were incubated for 30 min and washed with distilled water 5 times. To analyze the osteogenic differentiation, the samples were washed with the phosphate-saline buffer, and a 2% solution of Alizarin Red (pH 4.2) prepared with the phosphate-saline buffer was added. The samples were incubated for 30 min and thoroughly washed. To analyze the chondrogenic differentiation, the samples were washed with 1% acetic acid, and a 1% solution of Alcian Blue in acetic acid (pH 2.5) was added. The samples were incubated overnight at a temperature of 4° C, then washed with distilled water. Photoregistration of the stained samples was performed with an AxioVert.A1 light phase-contrast microscope (Carl Zeiss, Germany).

### Hydrogel system preparation

The applied hydrogel system was formed based on fibrin conjugates with PEG. To prepare the conjugates, homobifunctional PEG—O,O′-*bis*[2-(*N*-succinimidyl-succinylamino)-ethyl]polyethylene glycol (PEG-NHS; Sigma-Aldrich, Germany) was used. PEG-NHS was dissolved in a sterile phosphate buffer in the concentration of 1.5 mg/mL. The prepared PEG-NHS solution was added to a solution of fibrinogen (25 mg/mL) in the molar ratio of 5:1 (PEG-NHS: fibrinogen). The reaction was carried out for no less than 2 h at a temperature of 37 °C. The conjugates synthetized were routinely characterized using FTIR spectroscopy and had spectra similar to the typical ones previously published in our papers (the increased band at 1100 cm^−1^ which corresponded to the PEG-derived (C–O) units’ insertion) [39, 40, 42]. The gel was formed upon addition of an equal volume of thrombin with the concentration of 5 U/mL to the modified fibrinogen. Prior to the surgical intervention, the modified fibrinogen either with the added cell suspension or without the cell suspension and thrombin were kept in sterile single use 1.5 mL plastic tubes.

### Cell viability analysis

The assessment of the cells’ viability upon their encapsulation in a hydrogel system was performed with the use of Live/Dead staining and tests with AlamarBlue (Invitrogen, USA) and PicoGreen (Invitrogen, USA) [43]. A cell suspension in the concentration of 4.5 × 10^5^ cells per 1 mL of the gel was added to the gel and cultured for 24 h in the standard conditions. Then, staining was performed using a Live/Dead staining kit (Sigma-Aldrich, USA) and Hoechst 33258 (94403, Sigma-Aldrich, USA), and the samples were analyzed using an LSM 880 laser scanning confocal microscope equipped with an AiryScan module and a GaAsP detector (Carl Zeiss, Germany). The metabolic activity of cells was determined with the AlamarBlue test (Invitrogen, USA) according to the manufacturer’s instruction. The reagent was added to wells with the gel-encapsulated cells in the volume of 10%, and then, the plate was incubated for 2 h at 37 °C. The changes in the fluorescence intensity were measured at the wavelength of 590 nm with a Victor Nivo spectrofluorimeter (PerkinElmer, USA). The DNA concentration was measured using the PicoGreen test (Invitrogen) according to the manufacturer’s instruction, after the destruction of cell membranes by means of triple freezing–thawing and lysis of the gel with the proteinase K. An equal volume of the reagent was added to the lysate aliquots, and they were incubated for 5 min in the dark. The changes in the fluorescence intensity were measured at the wavelengths of 480 nm (excitation) and 520 nm (emission) with a Victor Nivo spectrofluorimeter (PerkinElmer, USA). A calibration curve was plotted to calculate the absolute DNA concentrations.

### In vivo experiments

In vivo experiments included two consecutive blocks of hetero- and orthotopic implantations and are described below in accordance with ARRIVE guidelines. The sample size (heterotopic implantation: 500 µL of the gel without or with 5 × 10^5^ cells per an animal, *n* = 24: 12 per condition; orthotopic implantation: 500 µL of the gel without or with 5 × 10^5^ cells or 500 µL of normal saline or 500 µL of MSC suspension per an animal, *n* = 24: 6 per condition) was determined based on our previous experiments with similar design, and the required number of relevant assessment methods and samples were also taken into an account [22, 44]. Inclusion and exclusion criteria were not applied due to the homogeneity of the experimental animals’ population at the start point of the study. In animal randomization, we used a random number generator with a given range to form groups. For bias and confounders minimization, blinding was applied on every stage. Groups were assigned with a code that eliminated unblinding, animals were assigned as a group code plus number in ascending order. Only during implantation, the surgeon could potentially distinguish solution and PEG-fibrin gel by higher viscosity. Implantations and measurements were made with alternation of animals and samples.

#### Heterotopic implantation

The subcutaneous implantation of the PEGylated-fibrin-based gel with and without cells was performed to male Wistar rats (3-month-old) with the weight of 300–400 g (*n* = 24: 12 in Group 1 (PEG-Fibrin) and 12 in Group 2 (PEG-Fibrin + MSC)). The analgosedation was performed by an intravenous injection of tiletamine-zolazepam and an intramuscular injection of xylazine solution based on 10–15 mg/kg and 1–2 mg/kg, respectively. A 3 × 3 cm region of skin at the animal’s withers was shaved, and the gel was administered subcutaneously using a 23G needle (500 µL of the gel without or with 5 × 10^5^ cells). No additional therapy was carried out in the post-surgical period. 3 and 7 days after the injection, 6 rats from each group were removed from the experiment by means of excessive intramuscular and intravenous administration of xylazine (0.5 mL, 1–2 mg/kg) and tiletamine-zolazepam (3 mL, 50 mg/mL + 50 mg/mL) solution, respectively, and the changes in the tissues surrounding the implant were visually assessed, and explants were harvested for the subsequent analysis.

#### Orthotopic implantation

**Fig. 1:**
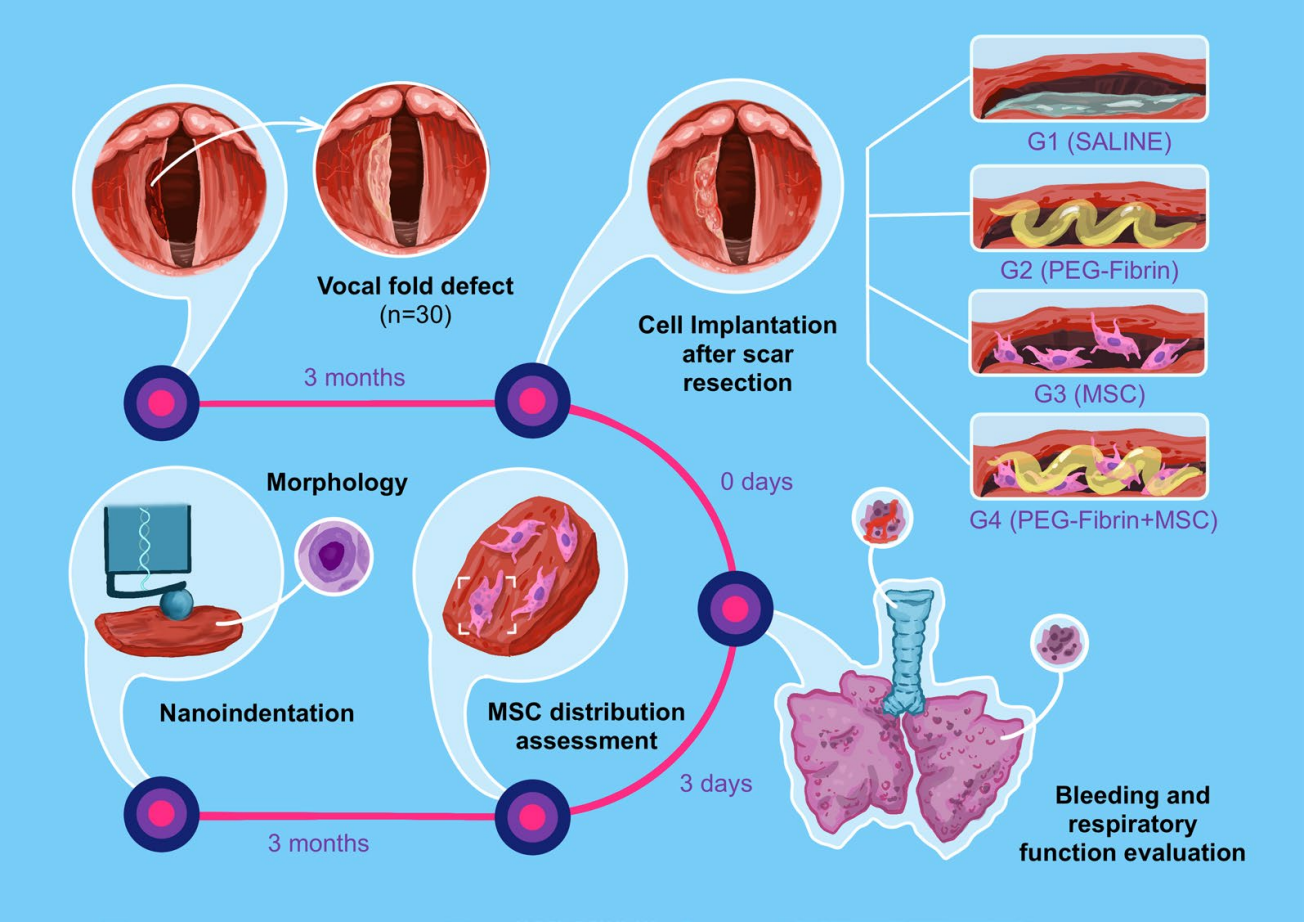
Experimental design. As tested agents, we chose a hydrogel system based on PEG-fibrin conjugates and a bioequivalent containing, beside the hydrogel, human bone marrow-derived MSC. The implantation was performed in the heterotopic (subcutaneously to rats) and orthotopic (secondary wound after the scar excision in rabbits) positions. In the first case, the animals were removed from the experiment on days 3 and 7, and a histological study was performed. In the second case, the VF scar was first modeled, it formed during 3 months, then, upon its excision, the cell-laden hydrogel system was administered, and the surgery efficiency was estimated 3 months later by means of histological and mechanical studies. The effects of the administered agents on the hemorrhage and breathing function were additionally studied, and the cells’ distribution in tissues was tracked depending on the used carriers. The figure is originally created by the authors

**Fig. 2:**
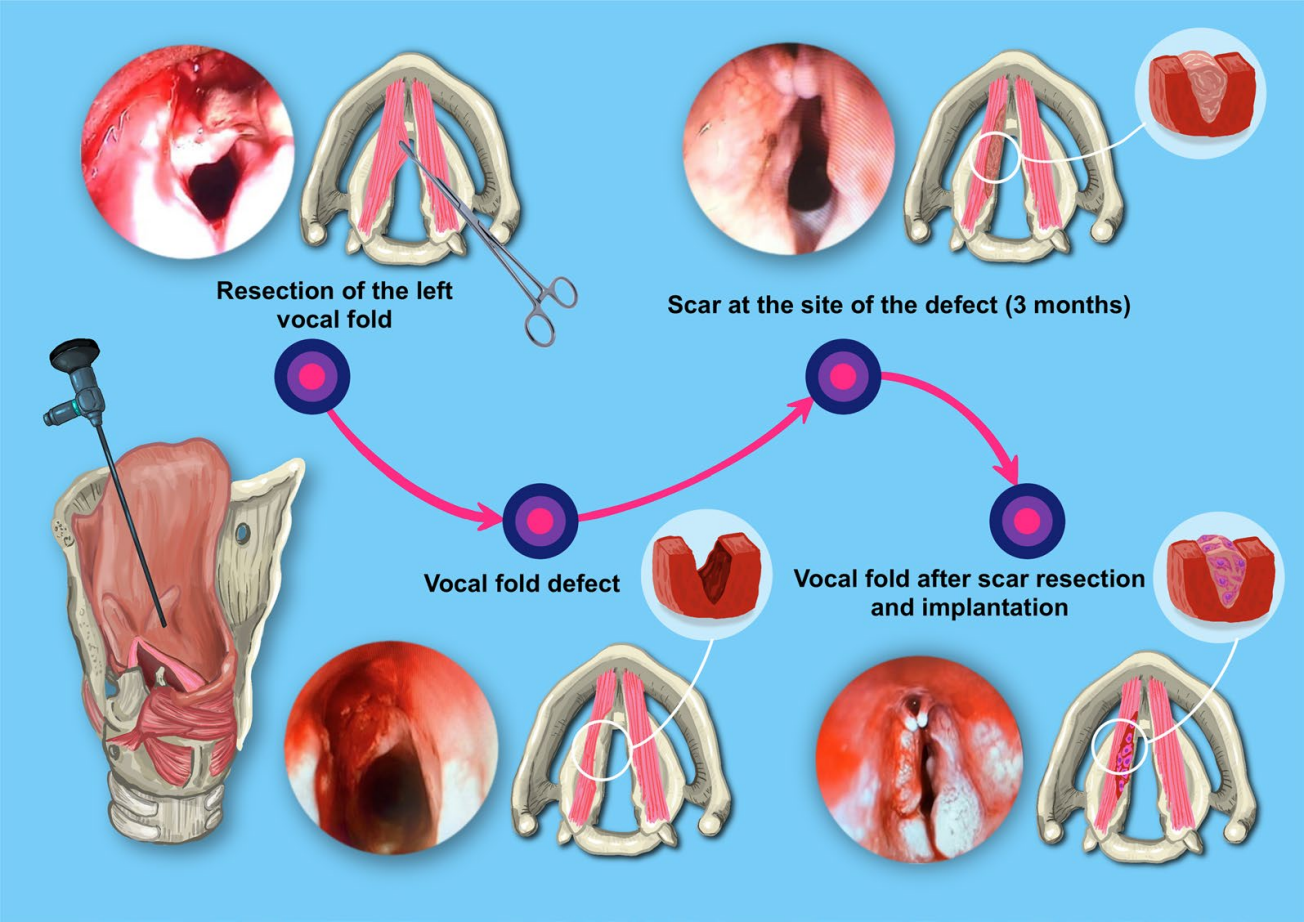
Stages of the scar formation in the rabbit VF and implantation: traction of the captured area of the left VF with forceps; VF defect; scar at the site of the defect; implantation of the PEG-fibrin gel and MSC. Endoscopy (0°)

The experiment was performed using male rabbits (“Sovetskaya Shinshila”, 8-month-old) with the weight of around 3000 g (*n* = 24). At the first stage, a scar on the VF was formed by surgical removal of a tissue fragment unilaterally; at the second stage (3 months post-surgery), the scar excision into a secondary wound was performed followed by the implantation. The following groups were formed (with 6 animals in each) depending on the agent administered to the operated VF: Group 1 (control)—normal saline, Group 2—PEG-Fibrin (without cells), Group 3—MSC suspension, Group 4—PEG-Fibrin + MSC (Figs. 1, 2).

To provide the painless manipulations and myorelaxation, analgosedation was performed by an intramuscular injection of tiletamine-zolazepam and a xylazine solution based on 10–15 mg/kg and 1–2 mg/kg, respectively. For the direct laryngoscopy of the larynx, a neonatal blade for orotracheal intubation, Miller C size 1, was used, and visualization was performed using a Karl Storz-Hopkins 0° 4.0 mm rigid endoscope. During the first stage, a unilateral defect of the left VF was created: the mucosa and the lamina propria of one-third of the VF were removed with the indent of 3–4 mm from the anterior commissure using cup forceps with the length of 18 cm and the grip surface diameter of 2 mm. When necessary, sanitation of blood clots and hemostasis were performed by pressing a cotton swab on a loop probe wetted with an adrenaline solution. Antibacterial therapy was applied in the post-surgical period: cefotaxime intramuscularly 75 mg/kg once per day, for 5 days.

In 3 months, the second surgery was performed, involving the VF scar identification followed by excision with cup forceps. The scar borders were determined with the visual and tactile assessment. Immediately afterward, the implantation was performed by an injection using a needle for endoscopic rhino-sinus surgery. The material (MSC suspension, PEG-Fibrin or PEG-Fibrin + MSC) was drawn in a 1 mL syringe, the “dead volume” of the needle was filled, after that the needle tip was inserted in the edge of the VF secondary wound, or actually in the mucosa’s margins around the defect and the lamina propria. The correct depth and site of injection were estimated by the formation of a characteristic infiltrate, the size of which correlated with the volume of the injected suspension. When using the gel, its components were mixed directly in the operation room. The implant volume was 500 µL that corresponded to 5 × 10^5^ cells in Groups 3 and 4.

The observation in the post-op period did not differ from that described above for the first surgery. No animal death due to complications was observed for all the cases. In the preset control times (3 months after the implantation, 6 months after the infliction of the defect), the animals were removed from the experiment by means of intramuscular and intravenous administration of xylazine (0.5 mL, 1–2 mg/kg) and tiletamine-zolazepam (3 mL, 50 mg/mL + 50 mg/mL) solution, respectively. Thus, the analysis was conducted 6 months after the experiment start and 3 months after the second wound healing.

#### Cell tracking after implantation

Visualization of the injected cells’ localization was performed in 3 days by the use of MSC with the stable expression of green fluorescent protein (GFP). To do this, cells were transduced using an LVT TagGFP2 recombinant lentivirus (Eurogen, Russia). Upon reaching the confluency of 80–90%, 50 µL of the suspension of viral particles (0.5 × 10^6^ transducing units/mL) and 10 µL of Polybrene were added to the cells, and the culture was incubated for 24 h at 37 °C in a CO_2_-incubator. On the next day, the culture medium was replaced. Upon reaching the confluency of 90%, the cells were detached, washed and sorted with a Sony SH800S cell microfluidic sorter (Sony Biotechnology, USA). The transduced MSC were injected in the form of a suspension (*n* = 3) or a gel (*n* = 3) into the site of scar excision in a rabbit. In 3 days, the tissues at the implantation site were excised, fixed, and embedded in OCT blocks. The sections were prepared using a cryotome and then, studied (up to 10 fields of view) using a laser scanning confocal microscope LSM 880 after additional staining of cell nuclei with Hoechst 33258 (94403, Sigma-Aldrich, USA).

#### Bleeding assessment

The intensity of the tissues’ intraoperative hemorrhage was estimated during the second surgery immediately after the implantation according to a 4-point scale: 0—minimal (traces of blood on the wound surface); 1—weak (the wound surface is covered with blood clots here and there); 2—moderate (the wound surface is completely covered with blood clots, but no blood accumulation is seen in the lumen of the larynx, no additional hemostasis is required); 3—pronounced (requiring aspiration of blood clots from the lumen of the larynx and application of a cotton swab on a loop probe wetted with an adrenaline solution pressed against the wound surface); 4—maximal (prolonged hemostasis is required by pressing a cotton swab on a loop probe wetted with an adrenaline solution to the wound surface, repeated aspiration of blood clots (Fig. 5A).

#### Breathing ability assessment

The severity of respiratory failure was assessed immediately after the second surgery by the following three parameters: intensity, duration of stridor and respiratory rate (RR). To assess the stridor intensity, a 3-point scale was used: 0—free breathing (on auscultation, the air passing through the glottis detected only by a phonendoscope and with auscultation from the anterior neck surface); 1—weak (noise, detected at a distance closer than 0.5 m); 2—moderate stridor (noise, detected at a distance of 1 m and farther); 3—pronounced (noise, diminished breath sounds). The presence of stridor was estimated every hour up to the total disappearance. RR was estimated as the number of chest wall excursions per minute 10 min after the surgery was completed.

#### Mechanical properties

To measure the local mechanical characteristics (Young’s modulus) of rabbit VF samples, a Chiaro nanoindenter (Optics11, Netherlands) was used. A probe with the spring constant of 0.05 N/m and sphere radius of 9.5 µm was utilized. Indentation was conducted in the phosphate-saline buffer medium at room temperature immediately after an animal’s removal from the experiment and the larynx dissection. Samples were immobilized at the bottom of a Petri dish with grips. During the measurement, the cantilever and attached optical fiber were always in the medium at a sufficient depth to prevent errors originated from the surface tension at the “air–water” boundary. 4 to 6 VF samples from each group were studied. 6 intact VF samples were additionally studied as a control. The area over which the Young’s moduli were measured was 1000 × 1000 µm with the step of 100 µm by both *X* and *Y* axes. The Young’s modulus was calculated from force–displacement curves according to the Hertz’s model of rigid sphere indenting an elastic half-space, using the Optics11 Dataviewer software. Based on the obtained data, the effective Young’s moduli (mean ± SD, kPa) of samples were calculated. After the measurements were completed, samples were dissected and placed in formalin.

The statistical analysis was performed with the Statsoft Statistica 64 software. The normality of distribution of the Young’s moduli data was estimated by the Kolmogorov–Smirnov test, and the homogeneity of variance was estimated by the Levene’s test. Due to the absence of normality in most distributions and inhomogeneity of dispersion inside the normal groups, the Mann–Whitney test was used to determine the statistical significance of the differences between the groups. The threshold of the statistical significance was set at *p* ≤ 0.05.

#### Histological analysis

The tissues at the implantation site were excised, fixed in 10% neutral buffered formalin for 24 h and embedded in paraffin blocks. Four-μm-thick sections of the formalin-fixed-paraffin-embedded tissue samples were stained with hematoxylin and eosin (H&E); orcein for the detection of elastic fibers; and with Picrosirius Red (PSR) for the detection of collagen fibers. A Leica DM4000 B LED microscope, equipped with a Leica DFC7000 T digital camera running under the LAS V4.8 software (Leica Microsystems, Wetzlar, Germany), was used for the examination and imaging of the samples. The specimens were studied by the methods of standard (for H&E, orcein, and PSR stained samples), phase-contrast (H&E) and polarized light (PSR stained samples) microscopies. The morphometric analysis of the histological samples was performed by two blinded pathologists. Any discrepancies in their results were resolved by a third pathologist. This pathologist had the knowledge of where the samples’ group belonged and wrote the histological report for each study group.

The morphometric analysis was conducted by 11 signs (area of the scar, epithelium hypertrophy, epithelium dystrophy, epithelium atrophy, elastic fibers, density and inhomogeneity of collagen fibers’ architecture, decrease in the fibroblasts’ cell-ness, infiltration and fibrosis of the muscle tissue, vascularization) with the use of a 4-point scale (0—absence of changes compared to the normal state; 1—weak changes; 2—moderate changes; 3—pronounced changes; 4—maximal changes) [22]. The calculations of the significant differences for the point-based estimation (semi-quantitative analysis) were performed with the GraphPad Prism version 7.00 for Windows (GraphPad Software, Inc.). The Kruskal–Wallis test was used for the search of differences, with the post hoc Dunn’s test for the pairwise analysis of the criteria between the groups. The level of statistical significance was set at *p* < 0.05. The multiple comparison correction was performed with the Benjamini–Hochberg procedure (false discovery rate).

The measurement of the scar’s and lamina propria’s thickness was performed with the Leica Application Suite Version 4.9.0 software at no fewer than 5 fragments located at a distance of 400 µm from each other. The measurements were conducted in a parallel way from the deep edge of the epithelial layer to the edge of the muscle tissue. The statistical analysis of the qualitative data was performed in the GraphPad Prism software version 7.00 for Windows (GraphPad Software, Inc.). The normal distribution of the data (tissue thickness) was estimated by the Shapiro–Wilk test. The differences between the groups were estimated using the ANOVA dispersion analysis and the Tukey test. The level of statistical significance was set at *p* < 0.05.

### Statistical analysis

When comparatively estimating the intensity of intraoperative hemorrhage and breathing failure, the data distribution was assessed with the Shapiro–Wilk test. Due to the data distribution differing from the normal one, the statistical analysis was performed using the nonparametrical single-factor Kruskal–Wallis test (for the estimation of differences between groups in total) and the Wilcoxon signed-rank test (for the search of differences between groups separately). The R software (version 3.6.1) was used for the calculations, and the ggplot2 library was used for the visualization of results. The multiple comparison correction was performed with the Benjamini–Hochberg procedure (false discovery rate).

## Results

### Characterization of MSC and the MSC-based bioequivalent

**Fig. 3:**
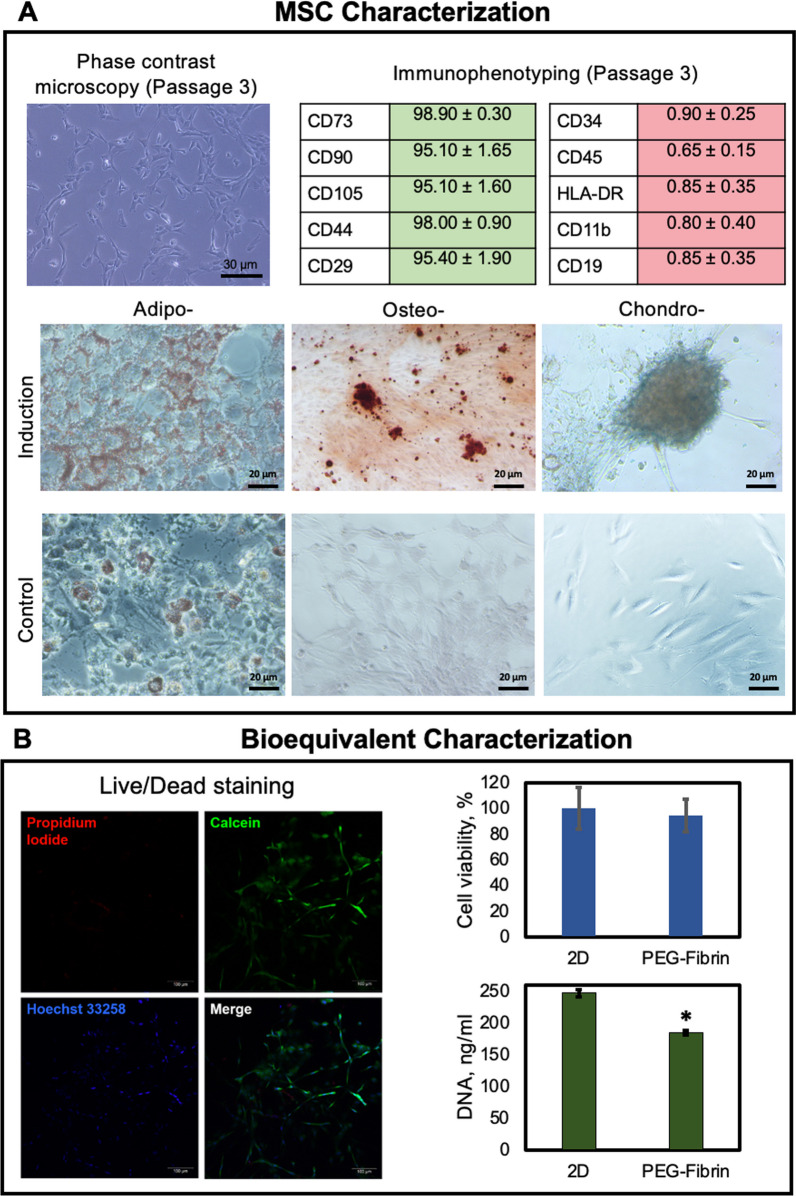
**A** MSC characterization (phase-contrast microscopy and immunophenotyping of the primary cell culture at Passage 3; cell differentiation into the three lineages (adipo-, osteo-, and chondrogenic); **B** Bioequivalent characterization (Live/dead staining: live cells—green fluorescence (Calcein AM), dead cells—red fluorescence (propidium iodide), nuclei—blue fluorescence (Hoechst 33,258), laser scanning confocal microscopy; cell viability measured using the AlamarBlue assay; total DNA content measured using the PicoGreen assay)

To confirm that the isolated cells belonged to mesenchymal stromal cells, we used the conventional set of methods [45]. The morphological analysis of the culture showed that the cells were attached to the culture plastic surface, had a typical shape, spread, actively migrated and proliferated forming a dense monolayer (Fig. 3A). The culture was capable of differentiation in the adipo-, osteo-, and chondrogenic directions (Fig. 3A). The analysis of the surface markers’ expression in the cell culture confirmed the mesenchymal nature of the cells (Fig. 3A, Additional file 1: Fig. S1). More than 95% of the cell population expressed classical surface markers such as CD90, CD73, CD105, CD44, CD29. A low level of expression for the CD11b, CD19, CD34, CD45, HLA-DR markers testified the absence of admixed endothelial, lymphocytic and leukocytic cells (the expression of those markers corresponded to the conventional level for an MSC population and did no exceed 0.90%).

To analyze the viability of the developed bioequivalent, qualitative (Live/Dead staining) and quantitative (AlamarBlue and PicoGreen) tests were used (Fig. 3B). It was shown that the cells remained viable inside the hydrogel system and retained their metabolic and proliferative activity. No dead cells were found by Live/Dead staining, and the whole construct’s viability did not differ from that in a monolayer culture.

### Heterotopic implantation

**Fig. 4:**
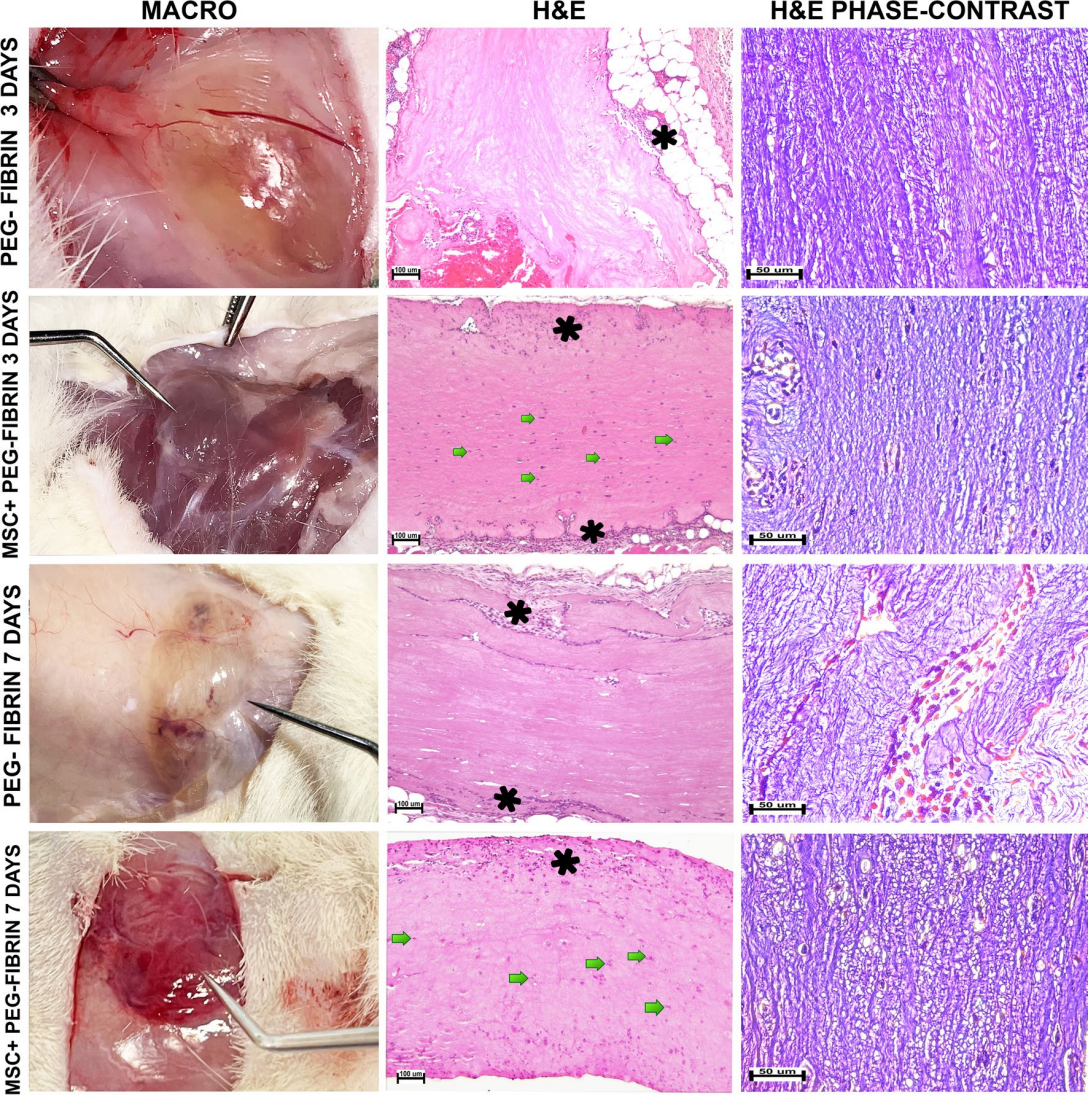
Heterotopic (subcutaneous) implantation of the bioequivalent to rats (days 3 and 7 after the surgery). Left: implants before excision (absence of reactive changes). Center: light microscopy, H&E stain, × 100; PEG-GEL 3 DAYS: a cell-less mass of an implant, surrounded by adipose tissue; PEG-GEL 7 DAYS: the marginal region of an implant is divided into strands, separated from each other by accumulations of cell elements; PEG-GEL + MSC 3 DAYS: regions of marginal resorption in the implant’s peripheral zone; PEG-GEL + MSC 7 DAYS: integral structure of an implant outside the muscle tissue, cells of the central and peripheral zones are seen, marginal resorption over the whole implant surface. Right: phase-contrast microscopy, H&E stain, × 400; PEG-GEL 3 DAYS: longitudinally extended fibrin fibers; PEG-GEL 7 DAYS: fibrous structure of the implant’s tissue, looser on day 7, the ingrowth of thin-walled capillaries is seen; PEG-GEL + MSC 3 DAYS: parallel orientation and compact packing of fibrin fibers; PEG-GEL + MSC 7 DAYS: fibrin fibers. Marginal regions (*) were infiltrated with lymphocytes (small oval mononuclear cells with a diameter of 8 µm) and macrophages (larger cells with U-shaped nucleus). Deeper tissues included groups of MSC (green arrows)

3 and 7 days post-implantation, the hydrogel system based on PEG-fibrin conjugates and the bioequivalent containing human bone marrow-derived MSC were found by the tactile assessment at the site of injection (subcutaneously). On the visual assessment, no reactive phenomena, exudate or pus accumulations were observed in the surrounding tissues. On both the 3rd and 7th days, the implants represented an integrated semi-transparent mass with clear contours of soft-elastic consistence, its size not significantly varying between the groups (Fig. 4). The implants could be separated from the tissues without ruptures and damage; however, they were transferred to the histological study with the complex of surrounding tissues with a 3–5-mm-wide margin.

On the third day after the surgery, the hydrogel system based on PEG-fibrin conjugates retained its integrated structure was surrounded by adipose tissue and a thin capsule. The phase-contrast microscopy study showed that the implant consisted of generally disordered, but, at some places, organized thin fibers (fibrin). Cells were not found inside the implant, only accumulations of erythrocytes. The inflammatory reaction around the implant was almost absent, and we observed only few macrophages and lymphocytes at the edges and almost completely absent resorption (biodegradation) that indicated a very low immune response to implantation (Fig. 4). In 7 days, the implant retained its integrated structure, and the inflammatory reaction was also absent. The difference with the 3d day was presented by the ingrowth of thin-walled blood vessels in the implant, as well as strands of granulation tissue consisting of fibroblasts, macrophages, lymphocytes, and numerous blood vessels. The presence of thin collagen fibers was revealed (connective tissue strands) which divided the gel to strata upon their ingrowth. There were few cell elements inside the implant, represented mainly by macrophages. No notable lysis was observed (Fig. 4). Thus, up to 7 days the implant does not undergo degradation and does not induce a noticeable inflammatory reaction that indicates its bioinertness.

The structure of the bioequivalent which included an MSC-laden hydrogel also preserved its integrity on the 3rd day. The fiber network did not differ from that for the implanted hydrogel alone without cells (phase-contrast microscopy showed fibrin fibers with parallel compact packing). Over the entire implant perimeter, fragments with marginal resorption were noted, filled with lymphocytes and macrophages. As compared to the hydrogel system without cells, lympho-macrophagal infiltration was more pronounced, starting from day 3. MSC in the gel bulk were spindle-shaped and oriented in parallel to the fiber structure and skin surface. On day 7, the implant was also determined in the space between the skin and back muscles. The peri-implant tissues were moderately infiltrated with immune cells, primarily, lymphocytes and macrophages. Infiltration was more pronounced than that on day 3. Marginal resorption was seen over the whole implant surface, which distinguished it from that observed on day 3, with the island-like character. A part of cells in the central zone was in a dystrophic state, no cytoplasm was found, and the nuclei were compact, polymorphic. The gel around such cells was vacuolized. Another part of cells was in the proliferative state, and the cells were large with big oval nuclei. For cells of the peripheral zone, dystrophic changes were not characteristic (Fig. 4).

### Orthotopic implantation

#### Assessment of the intensity of the intraoperative hemorrhage and breathing function

**Fig. 5:**
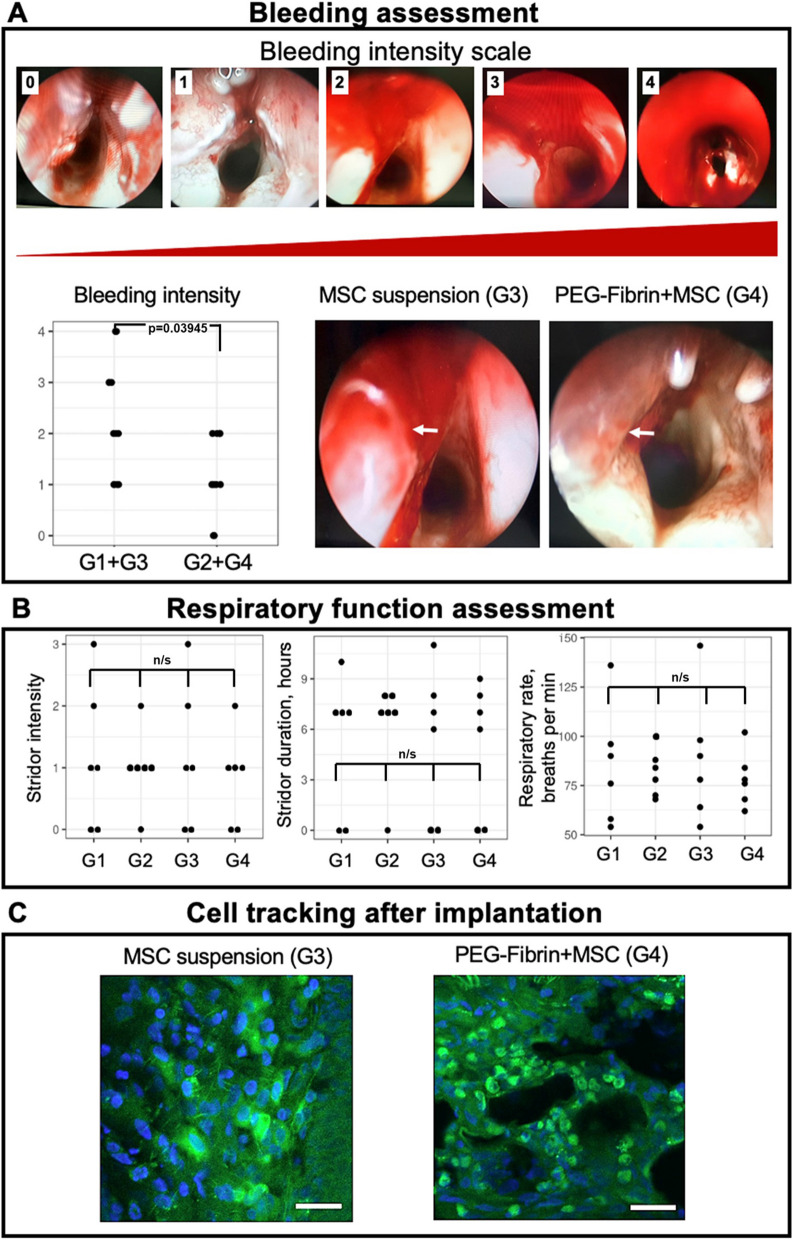
**A** Bleeding assessment (endoscopic images of intraoperative bleeding (endoscope 0°), a dot on the graph represents a value for each case, arrow points to the implantation site); **B** Respiratory function assessment (a dot on the graph represents a value for each case); **C** Cell tracking (transduced GFP-expressing MSC—green fluorescence; nuclei—blue fluorescence ((Hoechst 33,258), 3 days after implantation, scale bar—25 µm)

When comparing the hemorrhage intensity between the groups in total, no statistically significant differences were found (*p* = 0.2075). Statistically significant differences were absent between separate groups, as well (the *p*-value was from 0.37 to 0.87). However, when comparing the hemorrhage intensity between the combined groups with the hydrogel system based on PEG-fibrin conjugates and the control groups, statistically significant differences were revealed (*p* = 0.03945): the gel application was characterized by a lower hemorrhage intensity (Fig. 5A).

When comparing the intensity of stridor between the groups in total and between separate groups, the differences were not statistically significant (*p* = 0.96 and *p* = 1, respectively). No significant differences were observed in the stridor duration between the groups in total (*p* = 0.9497) and between separate groups (*p* = 1) (Fig. 5B). The RR comparison did not reveal any differences between the groups in total (*p* = 0.9593) and between separate groups (*p* = 0.97…1) (Fig. 5B). It should be noted at the same time that clear and statistically significant differences were observed in the stridor duration and RR for different stridor intensity (*p* = 0.00038 and *p* = 0.00049, respectively).

#### Cell tracking

The analysis of micropreparations with a confocal laser scanning microscope showed that the cells were present at the implantation site in 3 days. The cells were detected only in the tissues closely surrounding the VF defect. However, in the case of the bioequivalent a higher local MSC concentration was noted (intense green fluorescence) at the injection site (with studying up to 10 fields of view), as well as their migration in the thyroid-arytenoid muscle (Fig. 5C).

#### Histological analysis

**Fig. 6:**
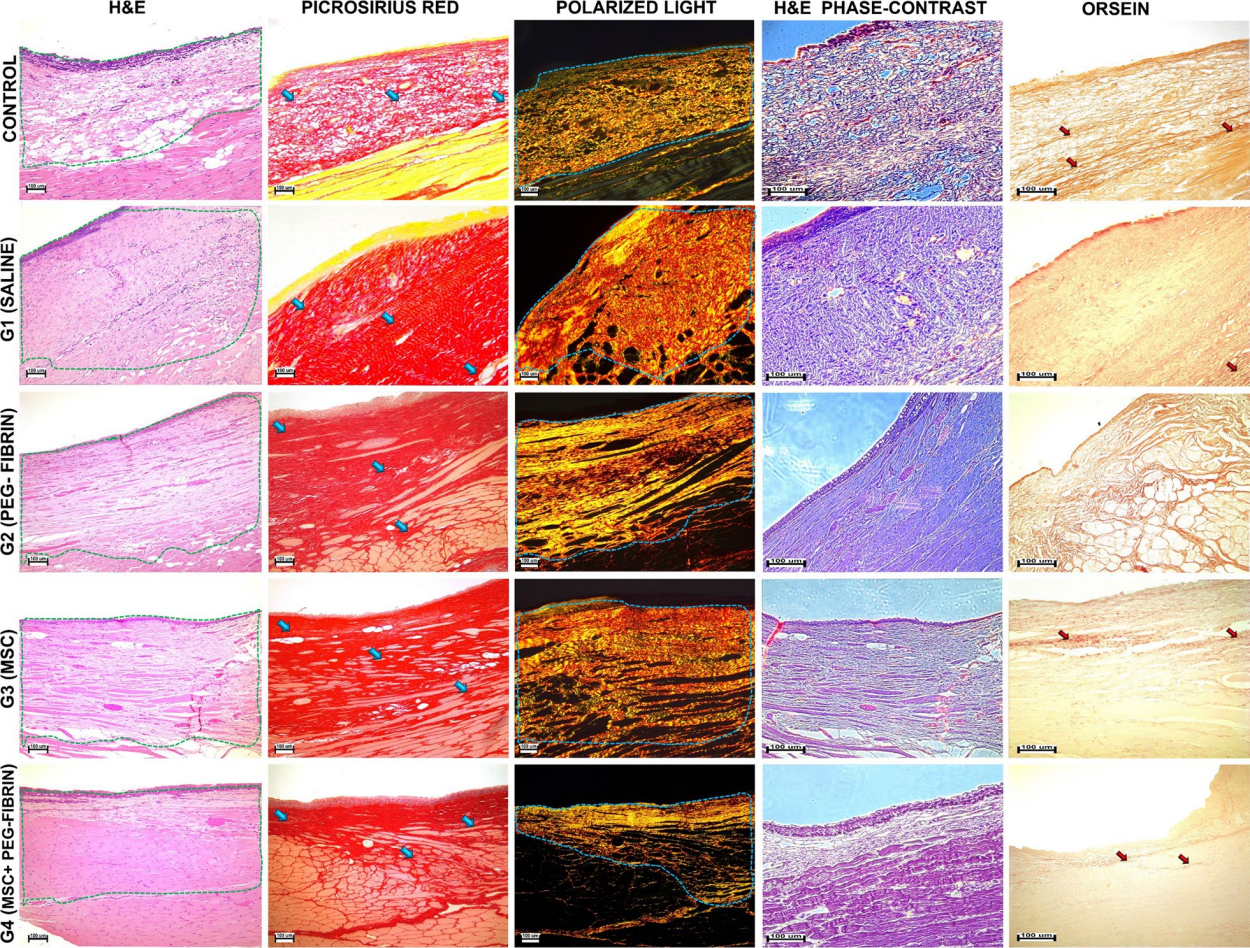
Orthotopic implantation to rabbits (Control—intact VF, G1—saline, G2—PEG-Fibrin, G3—MSC suspension, G4—PEG-Fibrin + MSC). Light microscopy, H&E stain, magnification × 100: Control—VF mucosa, a network of longitudinally oriented collagen fibers is clearly seen, in the inner layer they are looser and thinner than those in the outer layer; G1—vast scar, lined with multi-layered squamous epithelium, has a triple-layered structure; G2—thick scar, lined with multi-layered squamous epithelium, with muscle fibers inside; G3—scar, lined with multi-layered squamous epithelium, isolated muscle fibers remain inside; G4—thin scar, lined with multi-layered squamous epithelium, small inclusions of isolated muscle fibers are seen. Light microscopy, PSR stain, magnification × 100: Control—collagen fibers of the mucosa’s lamina propria (red); G1—pronounced fibrous-cicatricious changes of the VF mucosa; G2—scar consisting of densely packed collagen fibers; G3—relatively thick layer of scar tissue with bright-red staining of collagen fiber bundles; G4—bright-red collagen fibers of scar tissue coated with epithelium. Polarized light, PSR stain, magnification × 100: Control—collagen fibers have clear anisotropy, mainly of the yellow color; G1—pronounced anisotropy of densely packed collagen fibers; G2—collagen fibers have anisotropy with the red and yellow color; G3—pronounced anisotropy of collagen fibers; G4—anisotropy of collagen fibers is seen, mainly of the red and yellow color. Phase-contrast microscopy, H&E stain, magnification × 200: Control—a relatively loose network of longitudinally oriented collagen fibers is clearly seen; G1—dense scar tissues, the structure of collagen fibers comprising collagen bundles, crimps, is clearly seen; G2—scar consisting of densely packed collagen fibers, muscle tissue has numerous connective tissue interlayers; G3—relatively wide layer of scar tissue; G4—thin collagen fibers of a loose scar. Light microscopy, orcein stain, magnification × 200: Control—a network of elastic fibers is clearly seen in the mucosa; G1—total absence of elastic fibers in the scar tissue; G2—elastic fibers are absent in the scar; G3—elastic fibers are absent in the scar, a small number of elastic fibers is present in connective tissue interlayers growing in the muscles; G4—elastic fibers at the border of the scar and muscle sheath. Margins of the implantation area were lined with broken lines. Collagen fibers were marked with blue arrows, elastic fibers—black arrows

The intact VF’s structure in the control group was typical for the rabbit VF. In the central part, the VF was lined with multi-layered squamous epithelium, which changed to multi-row ciliary epithelium at the periphery. In the mucosa, weak lympho-macrophagal infiltration was seen here and there. The connective tissue of the mucosa in the VF’s central part was represented by relatively thin collagen fibers located longitudinally and in a parallel way to each other, forming two layers: a looser one—inner layer, closest to the epithelium, and a denser one—outer layer, visualized by staining with PSR and phase-contrast microscopy. Polarized light microscopy revealed anisotropy of collagen fibers. Elastic fibers formed thin bundles and were located both in a parallel and perpendicular direction in respect to collagen structures (Fig. 6).

In Group 1 (normal saline), in most animals the mucosa scars developed at the surgical defect site were lined with multi-layered squamous epithelium with dystrophic changes. The scars’ area was rather large, its structure included 3 layers. The superficial layer was represented by numerous spindle-shaped fibroblasts, located chaotically, similar to collagen fibers; the medium layer consisted of collagen fibers located more longitudinally; in the lower layer, the number of fibroblasts was diminished, collagen fibers were parallel to each other. The essential ingrowth of fibrous-cicatricious tissue in the muscle layer was noted. When stained with PSR, collagen fibers acquired a red color, were packed densely and chaotically in all layers (less pronounced in the superficial layer). The polarization color was mainly yellow and partially red. Phase-contrast microscopy allowed visualization of the structure and orientation of collagen fiber bundles. Elastic fibers were absent directly in the scar tissue, but few of them were found in the connective tissue interlayers ingrown in the muscles (Fig. 6).

In Group 2 (PEG-Fibrin), a scar tissue was formed at the site of the defect, lined with multi-layered squamous epithelium without noticeable dystrophic changes. It did not differ from that of Group 1 (normal saline) by its thickness and area. 3 months after the injection, the gel was not found. The scar tissue consisted of spindle-shaped fibroblasts located mostly along the surface. Collagen fibers were located densely along the scar. The connective tissue’s ingrowth in the muscle was noted. Weak diffuse infiltration with macrophages and lymphocytes was revealed. In some places, severely dilated blood vessels with erythrocytes in the sludge state were observed. The fibrous tissue density was clearly visualized by PSR staining, and the scar tissue had a pronounced anisotropy in polarized light. A red glow of fibers with a touch of yellow indicated the collagen fibers’ maturity. Phase-contrast microscopy clearly showed longitudinally located collagen fibers. No elastic fibers were found in the scar (Fig. 6).

In Group 3 (MSC suspension), a tendency to the reduced scar area was observed in comparison with the untreated scars; however, the differences did not reach the statistical significance (*p* = 0.07), and the scar tissue was looser. Some animals had scar tissue lined with epithelium with highly expressed atrophy and desquamation, and in some places, there was no epithelium. Other animals had multi-layered squamous epithelium with small dystrophic changes. The scar tissue was represented by two layers. The superficial (relatively narrow) layer was formed by fibroblasts, which, similar to collagen fibers, were located longitudinally. The deep layer (wide) was formed by the fibrous tissue, which grew in the muscles and isolated individual muscle fibers. When stained with PSR, collagen structures acquired a bright-red color. Polarized microscopy revealed pronounced anisotropy of collagen bundles. Phase-contrast microscopy clearly visualized the structure of the scar tissue’s collagen fibers and isolated muscle fibers inside the scar. Elastic fibers were absent in the scar, and a few of them were found in the connective tissue interlayers ingrown in muscle fibers (Fig. 6).

In Group 4 (PEG-Fibrin + MSC), cicatricious changes of the mucosa were found at the post-surgical defect site. The scars were lined with multi-layered squamous epithelium with a well-defined arrangement of layers, almost indistinguishable from the normal mucosa epithelium. The gel was not found after 3 months. The scars were relatively narrow in their thickness, not wider than the intact mucosa. Some animals had inclusions of individual muscle fibers in the scar, and collagen fibers and fibroblasts had a mainly longitudinal orientation. Other animals had loose scar tissue, collagen fibers were intertwined, and fibroblasts were located without order. When stained with PSR, collagen fibers were colored red with the same intensity as they had in the intact mucosa. Polarized light microscopy revealed anisotropy of red-colored collagen fibers with large inclusions of the yellow color. Phase-contrast microscopy clearly visualized thin collagen fibers in the loose scar tissue. Elastic fibers were identified at the border of the scar and muscle sheath (Fig. 6).

**Fig. 7:**
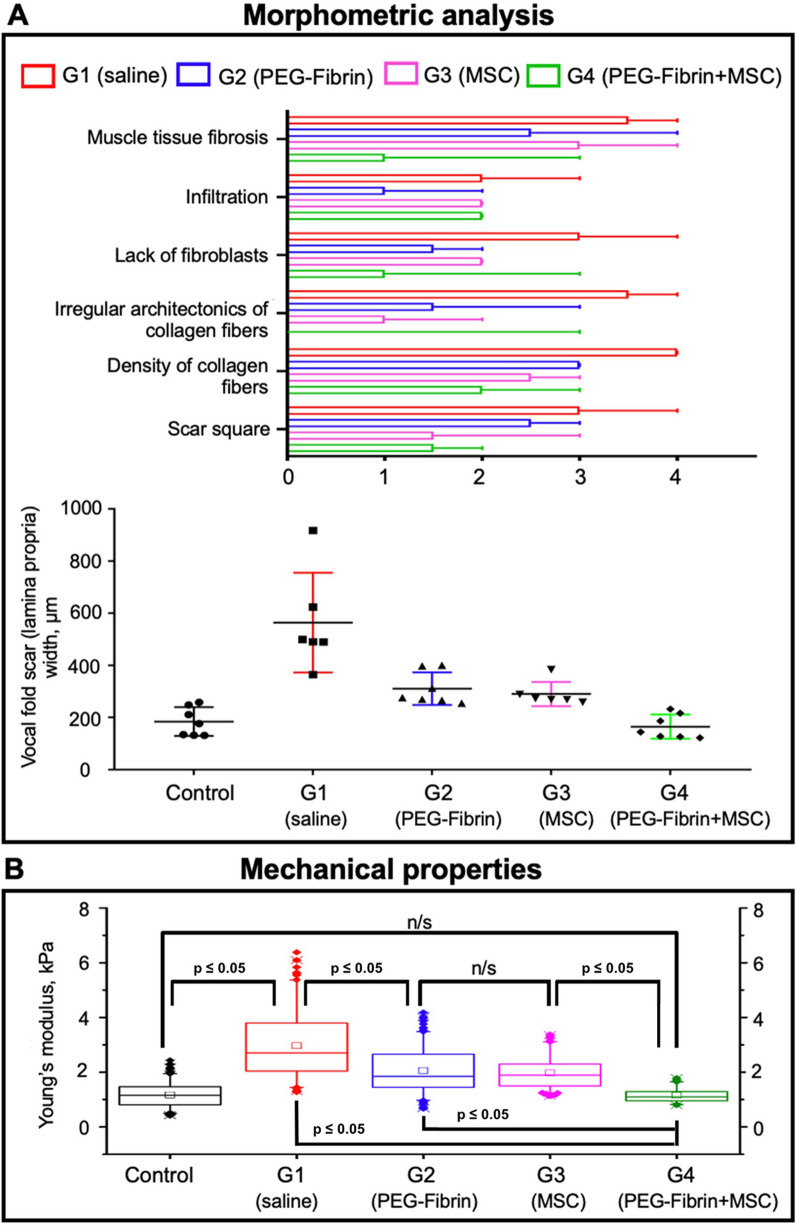
**A** Morphometric analysis (parameters with a significant difference in values, median ± 95%CI; lamina propria—control, scar—G1-G4, a black symbol shows a value for one sample, mean values ± SD); **B** Mechanical properties of the untreated and treated VF (Young’s modulus (kPa), nanoindentation in a liquid medium; n/s—not significant)

The statistical analysis of the morphometric data revealed that, in Group 4 (PEG-Fibrin + MSC), collagen fibers of the scar were packed looser, at the same time they were more frequently mutually parallel, that is inherent in the normal mucosa structure. In Group 1 (normal saline), fragments with chaotic intertwined packing of fibers prevailed, and we observed the formation of crimps—regions with wavy, dense and parallel packing of collagen, characteristic of tendons (Fig. 7A). These findings are reflected in the statistically significant differences between the groups by the criteria of the architecture irregularity and density of collagen fibers (*p* = 0.018 and *p* = 0.048, respectively) (Fig. 7A). A tendency to the reduction in the intensity of these criteria-based changes should be noted for Group 3 (MSC suspension), as well. However, the differences with Group 1 (normal saline) did not reach the statistical significance: *p* = 0.228 for the criterion of the collagen fibers’ irregularity, and *p* = 0.06 for the density. In all the experimental groups, we observed the fibrous tissue’s ingrowth in the adjacent exterior muscle tissue; however, in Group 4 (PEG-Fibrin + MSC), the muscle tissue fibrosis was much less pronounced than it was in Group 1 (normal saline) (*p* = 0.008) (Fig. 7A). The content of fibroblasts in Group 1 (normal saline) was the lowest, the differences appeared significant when comparing with Group 2 (PEG-Fibrin) (*p* = 0.03), while the differences with Group 4 (PEG-Fibrin + MSC) were close to the threshold of significance (*p* = 0.07) (Figs. 6, 7A). According to the criteria of infiltration, vascularization and content of elastic fibers in the scar, no statistically significant differences were found between the groups.

The results of the thickness measurements for the scar tissue and lamina propria formed a normal distribution. The highest thickness was found in Group 1 (normal saline), with the average value of 564.3 ± 191.6 µm. In Groups 2 (PEG-Fibrin), 3 (MSC suspension) and 4 (PEG-Fibrin + MSC), the average thicknesses of the scars were lower: 311.1 ± 62.66 µm, 290.5 ± 46.63 µm, and 165.2 ± 46.19 µm, respectively. The average thickness of the lamina propria in the intact VF was 184.7 ± 55.25 µm. The statistical analysis showed significant differences in the scar thickness between Groups 2 (PEG-Fibrin), 3 (MSC suspension) and 4 (PEG-Fibrin + MSC) upon their pairwise comparison with Group 1 (normal saline), with the highest differences observed for Group 4 (*p* < 0.0001). The statistically significant differences between the thicknesses of the lamina propria in the control group and in Group 4 were absent (*p* = 0.995) (Fig. 7A).

#### Mechanical properties

The Young’s modulus of the intact VF was 1.17 ± 0.45 kPa, that is 2.4–2.6 times lower than the Young’s modulus of the VF scars (Group 1 (normal saline)), equal to 2.97 ± 1.22 kPa; the differences are statistically significant (*p* ≤ 0.05) (Fig. 7B). The Young’s moduli in the group treated with the hydrogel system only (Group 2(PEG-Fibrin)) were within 2.06 ± 0.81 kPa, that was statistically significantly higher than the moduli of the intact VF, but lower than those in Group 1 (*p* ≤ 0.05) (Fig. 7B). In the group where MSC were implanted as a suspension (Group 3 (MSC suspension)), the average Young’s modulus was 1.97 ± 0.55 kPa, that was significantly higher than the values for the intact VF (*p* ≤ 0.05), but significantly lower than the moduli in the scar group (*p* ≤ 0.05); no significant differences were found when comparing to the values in Group 2 (PEG-Fibrin) (Fig. 7B). The Young’s modulus of the VF after the bioequivalent implantation (Group 4 (PEG-Fibrin + MSC)) (1.15 ± 0.25 kPa) did not statistically significantly differ from the intact VF modulus (1.17 ± 0.45 kPa); therefore, the tissue properties in this group more closely resembled the intact VF. At the same time, the Young’s modulus values in Group 4 (PEG-Fibrin + MSC) were significantly lower when compared to other Groups (Group 1 (normal saline), Group 2 (PEG-Fibrin), Group 3 (MSC suspension)) (*p* ≤ 0.05) (Fig. 7B).

## Discussion

Our study on the regeneration potential of a bioequivalent consisting of a hydrogel system based on PEG-fibrin conjugates and human bone marrow-derived MSC was performed using a model of cicatricious lesions of the VF. The model implied two stages: at the first stage, a VF defect was formed by the 1/3 VF resection; at the second stage, 3 months after the first surgery, the bioequivalent was implanted by an injection into the secondary wound after the scar excision. Such as approach, despite being more laborious as compared to implantation in the intact VF defect, is more relevant to clinical practice, when physicians encounter chronic damage and recommend the surgical treatment no earlier than 6 months post-injury [15].

Nevertheless, no single opinion exists on the order of administration of such products: directly in the scar tissue or in the secondary wound after the preliminary scar resection. On the one hand, the fibrous tissue excision is an accepted approach to the treatment of laryngeal scars necessary in the case of their stenosing forms; on the other hand, such actions may pose a risk of a systematic error when estimating the results, and even adversely affect the final effect of therapy due to the additional defect expansion [26, 46]. In this study, the second approach was chosen, namely, the scar excision, in order to reduce the operation duration. We performed the scar tissue removal together with the epithelium, while in clinical practice the scar resection is performed submucosally. Nevertheless, such actions have a more traumatizing character due to more severe scarring as compared to a single injury.

After the implantation of human MSC in the rabbit VF, we did not apply immunosuppressive therapy, based on the data about low immunogenic and essential anti-inflammatory MSC properties [47, 48]. Kim et al. followed the same tactics when implanting human adipose tissue-derived MSC in the rabbit VF and did not note development of significant immune reactions [49–51]. Moreover, in experiments on heterotopic implantation, we revealed that MSC injected as a part of the bioequivalent had induced the low inflammatory reaction and were well tolerated.

The idea of a carrier for the injected cells is not new; however, gels based on hyaluronic acid and collagen have gained the largest acceptance [23, 24, 34, 50, 52–57]. Their application reduced inflammation, overall level of fibrosis, collagen deposition and scar thickness, and contributed to the restoration of the content of elastic fibers, hyaluronic acid, and other extracellular matrix components. Shiba et al. conducted a study on the use of fibrin-based carriers in vivo. They successfully performed implantation of a bioequivalent based on adipose tissue-derived MSC and fibrin in an acute wound of the rabbit VF and showed that the restored tissue did not undergo noticeable structural changes and could support vibration [58, 59]. In both cases, the authors used the constructs requiring suturing at an implantation site through the median laryngofissure. The results achieved are of particular interest for the development of a tissue engineering based strategy to treat extensive VF defects (including their total resection and laryngeal-tracheal cicatricial stenoses). Here, we injected the fabricated equivalent into the VF. This approach enables the precise endolaryngeal implantation into foci of interest almost without an influence on the native tissues. Therefore, patients with circumscribed VF defects will be less harmed because of no need to fixate a graft and less invasive surgery that could significantly reduce the duration of their rehabilitation.

The MSC survivability after systematic administration usually does not exceed 1% in one week; therefore, the main mechanism of their effects is considered to be mainly paracrine [49]. When implanted into the VF, they almost cannot be observed in 1–6 months. For instance, Svensson B. et al. showed that after implantation into an acute VF injury the survivability of bonemarrow-derived MSC was 0.18% in four weeks [60]. Hiwatashi N. et al. also revealed in one month, adipose- and bonemarrow-derived MSC did not remain at an implantation site; however, the use of a collagen carrier promoted their retention up to one month, but not longer than six months [28, 31]. Nevertheless, several researchers found that some implanted cells could differentiate, e.g., into fibroblasts, myofibroblasts, etc. [23, 61, 62].

According to our data on both hetero- and orthotopic implantations, the hydrogel system we applied did not induce any inflammatory reaction and promoted the cells’ survival and migration due to the formation of a favorable microenvironment (that correlates with the data obtained earlier in vitro [40, 63]) and due to the hemostatic effect preventing their washout during the intraoperative bleeding (cell tracking analysis in 3 days after surgery showed that MSC implanted within the gel were more concentrated in the treated tissues than those injected as a suspension). Therefore, fibrin gel retarded the cell migration from an implantation site during first three days. Such an effect from the hydrogel application for the treatment of VF defects was noted by Hertegård et al., who administered human bone marrow-derived MSC using a hyaluronic acid-based gel as a carrier and showed in vitro that encapsulated cells had the decreased migration ability [64].

In comparison with other Groups, the revealed regenerative potential of the fabricated bioequivalent might be explained by shifting a MSC expression profile as it is known, for instance, that higher amounts of MCP-1 were shown in the secretome of 3D cultures of bone marrow-derived MSC [64, 65] and fibrin gel can stimulate the synthesis of factors by MSC (e.g., VEGF, TGF-*α*, and IP-10) [38, 66, 67], which are the key players in wound healing and enhance the reparation of the damaged epithelium and angiogenesis [68].

It is of importance that no significant differences between the groups were obtained from the analysis of intensity, duration of stridor and RR, that indicated the absence of a higher risk of breathing failure as compared to the injection of an equal volume of normal saline. It should be emphasized that the assessment of hemorrhage and breathing failure has not been earlier performed in the experimental studies on the VF restoration with such techniques. However, this is important due to the possibility of loss of the implanted cells from the implantation site because of bleeding and a risk of reactive edema and implant migration.

The VF’s mechanical parameters directly affect the vocal characteristics; therefore, they are of particular attention in many studies. Parallel rheometry is a technique dominating by the frequency of application [20, 21, 23, 60, 69, 70]. For instance, in the studies by Svensson et al. and Kim et al. it was shown by shear rheometry that the repair of VF lesions by administration of bone marrow-derived MSC allowed bringing the mechanical properties (dynamic viscosity and shear modulus) of the restored tissue to the values close to those of the intact tissue [21, 70]. In spite of the fact that parallel rheometry allows for obtaining statistically significant differences in those parameters, it implies studying a microsample without taking into account the tissue surface. At the same time, a number of studies highlight an essential contribution of collagen fibrils’ microarchitecture to the VF’s mechanical properties and their impairment upon scarring [71–73]. Earlier, we have shown a possibility of atomic force microscopy application for the solution of this problem [22]. However, since the analysis was performed with the use of fixed (not native) samples, the obtained Young’s modulus differences did not reflect the absolute mechanical properties of the tissue. In this connection, in this study we used the technique of nanoindentation in a liquid medium. The Young’s modulus, reflecting the tissue stiffness, in all the post-implantation groups was significantly lower than that in the control group that indicates the formation of a more distensible tissue in the defect region. In Group 4 (PEG-Fibrin + MSC), the Young’s modulus was significantly lower in comparison with other experimental groups and did not statistically significantly differ from the values for the intact VF that testified the restoration of the VF’s properties.

## Conclusions

According to the results of the performed study, the bioequivalent consisting of a hydrogel system based on PEG-fibrin conjugates and human bone marrow-derived MSC allowed reducing the hemorrhage intensity during the surgery without increasing the risk of breathing failure. The probability of a critical drop in the cell concentration in the defect region was correspondingly decreased that, in general, improved the outcome of the damaged VF therapy at the observation endpoint. With the bioequivalent application, the VF tissue forming at the defect site was close to the native tissue in its structure and biomechanical properties.

### Supplementary Information

**Additional file 1. Figure S1**: Immunophenotyping the MSC culture (representative graphs achieved using flow cytometry).

## Data Availability

All data generated or analyzed during this study are included in this published article.

## Abbreviations

bFGF: Basic fibroblast growth factor
FBS: Fetal bovine serum
GFP: Green fluorescent protein
H&E: Hematoxylin and eosin
MSC: Mesenchymal stromal cells
PEG: Polyethylene glycol
PFA: Paraformaldehyde
PSR: Picrosirius red
RR: Respiratory rate
VF: Vocal folds
