# Supplementary material for: Vocal fold restoration after scarring: biocompatibility and efficacy of an MSC-based bioequivalent
Source: Stem Cell Res Ther. 2023 Oct 21;14:303. doi: 10.1186/s13287-023-03534-x (PMC10590531; doi:10.1186/s13287-023-03534-x)
Supplement: Supplementary file 1 — Additional file 1. Figure S1: Immunophenotyping the MSC culture (representative graphs achieved using flow cytometry). [file 13287_2023_3534_MOESM1_ESM.docx]

**Supplementary Materials**

**Vocal fold restoration after scarring: Biocompatibility and efficacy of an MSC-based bioequivalent (Svistushkin et al.)**


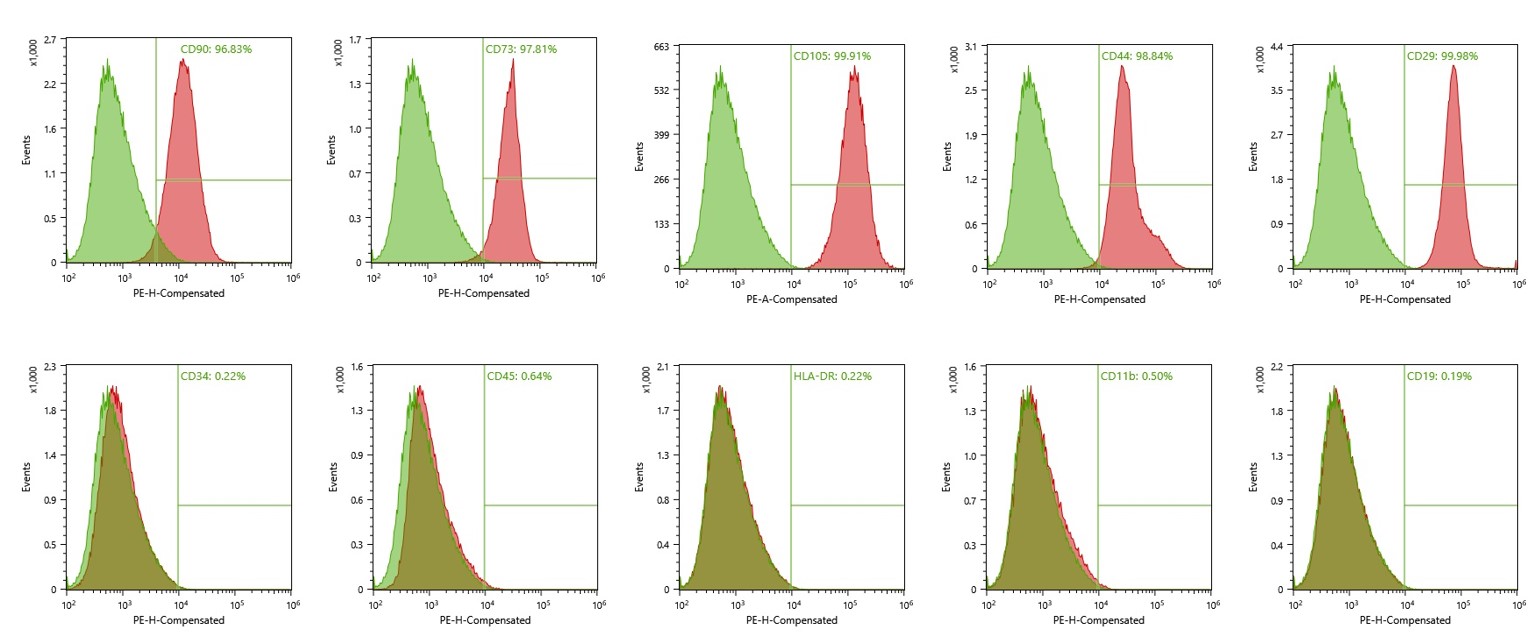


**Figure S1.** Immunophenotyping the MSC culture (representative graphs achieved using flow cytometry).
